# The Transmembrane Receptor TIRC7 Identifies a Distinct Subset of Immune Cells with Prognostic Implications in Cholangiocarcinoma

**DOI:** 10.3390/cancers13246272

**Published:** 2021-12-14

**Authors:** Thomas Albrecht, Benjamin Goeppert, Fritz Brinkmann, Alphonse Charbel, Qiangnu Zhang, Johannes Schreck, Nina Wilhelm, Stephan Singer, Bruno C. Köhler, Christoph Springfeld, Arianeb Mehrabi, Peter Schirmacher, Anja A. Kühl, Monika N. Vogel, Holger Jansen, Nalân Utku, Stephanie Roessler

**Affiliations:** 1 Institute of Pathology, Heidelberg University Hospital, Im Neuenheimer Feld 224, 69120 Heidelberg, Germany; Thomas.Albrecht@med.uni-heidelberg.de (T.A.); Benjamin.Goeppert@med.uni-heidelberg.de (B.G.); Fritz.Brinkmann@med.uni-heidelberg.de (F.B.); Alphonse.Charbel@med.uni-heidelberg.de (A.C.); qiangnu.zhang@gmail.com (Q.Z.); Johannes.Schreck@med.uni-heidelberg.de (J.S.); Peter.Schirmacher@med.uni-heidelberg.de (P.S.); 2 Liver Cancer Center Heidelberg (LCCH), 69120 Heidelberg, Germany; bruno.koehler@nct-heidelberg.de (B.C.K.); Christoph.Springfeld@med.uni-heidelberg.de (C.S.); Arianeb.Mehrabi@med.uni-heidelberg.de (A.M.); 3 Tissue Bank of the National Center for Tumor Diseases, Heidelberg University Hospital, 69120 Heidelberg, Germany; Nina.Wilhelm@med.uni-heidelberg.de; 4 Institute of Pathology and Neuropathology, Eberhard-Karls University, 72076 Tübingen, Germany; Stephan.Singer@med.uni-tuebingen.de; 5 Department of Medical Oncology, National Center for Tumor Diseases, Heidelberg University Hospital, 69120 Heidelberg, Germany; 6 Department of General, Visceral and Transplantation Surgery, Heidelberg University Hospital, 69120 Heidelberg, Germany; 7 Charité-Universitätsmedizin, Corporate Member of Freie Universität Berlin and Humboldt-Universität zu Berlin, iPATH.Berlin, 12203 Berlin, Germany; Anja.Kuehl@charite.de; 8 Diagnostic and Interventional Radiology, Thoraxklinik at Heidelberg University Hospital, 69126 Heidelberg, Germany; Monika.Vogel@med.uni-heidelberg.de; 9 Institute for Medical Immunology, Campus Virchow, Charité, Augustenburger Platz 1, 13353 Berlin, Germany; holger.jansen@cellact.eu

**Keywords:** immunotherapy, TIRC7, cholangiocarcinoma, immune checkpoint inhibitors, PD-L1, biomarkers, tumor microenvironment

## Abstract

**Simple Summary:**

Cholangiocarcinoma (CCA) is an epithelial malignancy with a dismal prognosis due to rapid tumoral spread and poor therapeutic options. Innovative therapies, such as immune-modulating drugs, are urgently needed. Here, we assessed the quantity and clinical implications of immune cells expressing the T cell immune response cDNA 7 receptor (TIRC7) in a cohort of 135 CCA patients. We found that TIRC7^+^ immune cells are present both in the tumor epithelia and in the stroma in the majority of patients with particularly high intraepithelial levels in intrahepatic CCA. Distinct histomorphological tumor subtypes were exclusively associated with a TIRC7^+^ phenotype. By correlation analysis, we uncovered that a high level of intraepithelial TIRC7^+^ immune cells was associated with a favorable prognosis in intrahepatic CCA. Our results reveal a distinct subset of immune cells marked by TIRC7 to be present and prognostically relevant in CCA.

**Abstract:**

Cholangiocarcinoma (CCA) is a heterogeneous malignancy with a dismal prognosis. Therapeutic options are largely limited to surgery and conventional chemotherapy offers limited benefit. As immunotherapy has proven highly effective in various cancer types, we have undertaken a quantitative immunohistopathological assessment of immune cells expressing the immunoinhibitory T cell immune response cDNA 7 receptor (TIRC7), an emerging immunoinhibitory receptor, in a cohort of 135 CCA patients. TIRC7^+^ immune cells were present in both the tumor epithelia and stroma in the majority of CCA cases with the highest levels found in intrahepatic CCA. While intraepithelial density of TIRC7^+^ immune cells was decreased compared to matched non-neoplastic bile ducts, stromal quantity was higher in the tumor samples. Tumors exhibiting signet ring cell or adenosquamous morphology were exclusively associated with an intraepithelial TIRC7^+^ phenotype. Survival analysis showed intraepithelial TIRC7^+^ immune cell density to be a highly significant favorable prognosticator in intrahepatic but not proximal or distal CCA. Furthermore, intraepithelial TIRC7^+^ immune cell density correlated with the number of intraepithelial CD8^+^ immune cells and with the total number of CD4^+^ immune cells. Our results suggest the presence and prognostic relevance of TIRC7^+^ immune cells in CCA and warrant further functional studies on its pharmacological modulation.

## 1. Introduction

Cholangiocarcinoma (CCA) represents a group of biologically heterogeneous malignant epithelial tumors arising either from the intrahepatic (iCCA) or extrahepatic (eCCA) biliary tract, with eCCA being further classified by either proximal (pCCA) or distal (dCCA) location [1,2]. CCAs most often present as aggressive, therapy-resistant adenocarcinomas characterized by early distant metastasis preventing surgical management [1]. Though recent studies provide new information on the molecular features of CCA, prognosis is overall still dismal with a median survival of less than one year [3]. Most patients are not eligible for resection at the time of diagnosis as symptoms tend to first emerge late in the course of the disease [4]. Palliative chemotherapy often remains the only therapeutic medical option. Considering the recent successes in targeted cancer therapies, innovative approaches are warranted for CCA patients.

Cancer was long considered to be the result of unlimited, invasive growth of autonomously acting cells that underwent malignant transformation due to distinct genetic alterations [5]. Thus, medical strategies focused on the tumor cells with drugs designed to suppress cell proliferation with conventional chemotherapy being the mainstay for most advanced malignancies [6]. The focus has changed with an appreciation of the determinative role of the complex tumor microenvironment on tumor progression [7,8]. In CCA and other tumor entities, the tumor microenvironment consists of a variety of different T cell subsets, endothelial cells, fibroblasts and other immune cells, which interact with each other and tumor epithelia to affect the behavior of the malignancy. [9]. CCA has a dense and desmoplastic tumor stroma. The ability of the tumor microenvironment to control cancer progression has been leveraged by checkpoint inhibitors such as anti PD-1 or anti CTLA-4-directed antibodies [10,11,12]. In solid tumors, such as metastatic melanoma, NSCLC and genitourinary cancers immunotherapy has altered therapeutic management and opened a new era in oncology [13,14].

Recently, we demonstrated that immune cell infiltration is a prognostic factor of CCA patient outcome [15]. CCA patients with intraepithelial tumor-infiltrating CD4^+^, CD8^+^ and FOXP3^+^ T lymphocytes showed significantly longer overall patient survival [15]. This points to the importance of understanding the composition of immune cell infiltrates in CCA. The expression patterns of factors important for T cell mediated immune therapies, such as PD-L1 and CTLA-4, may distinguish patient subgroups responsive to specific immune therapies. The transmembrane protein T cell immune response cDNA 7 (TIRC7) is expressed by activated T lymphocytes and has been implicated in control of T cell activation [16]. The regulation of immune activation is achieved via the binding of TIRC7 to its ligand HLADR-alpha2 domain [17]. TIRC7 signaling inhibits immune activation via the induction of CTLA-4 and caspase 7 and 9 and inhibition of Th1 cytokines in lymphocytes [17]. In contrast, TIRC7-deficiency results in increased proliferation and enhanced activation of immune cells via downregulation of CTLA-4, phosphorylation of STAT3 and enhanced cytokine secretion including interferon-γ [17,18,19]. Specific agonist TIRC7 antibodies have proven effective in animal models of immune-mediated diseases, e.g., kidney or heart transplant rejection, collagen-induced arthritis and acute graft-versus-host disease [20,21,22,23]. However, the expression of TIRC7 in tumor-infiltrating immune cells and the relevance of TIRC7 for cancer development or progression are still unclear. Therefore, we analyzed TIRC7 expression in a comprehensive, well-characterized cohort of CCA patients and assessed its impact on clinicopathological variables.

## 2. Materials and Methods

### 2.1. Clinicopathological Characteristics

Tissue samples were obtained from patients undergoing surgery for CCA at Heidelberg University Hospital between 1995 and 2010. Inclusion of tumor tissue for this study was approved by the institutional ethics committee (S-206/2005 and S-519/2019). Only patients with primary adenocarcinomas or adenosquamous carcinomas and without other known malignancies at the time of diagnosis were included in this study. Patients who received radiochemotherapy prior to surgery were excluded. The mean age of enrolled patients was 62.3 years and the median age 63.5 years. Approximately two thirds (65.9%) of all patients were male. Tumors were restaged according to the 8th edition of the TNM Classification of Malignant Tumors and classified based on the World Health Organization tumor classification system [24]. In total, the cohort consisted of 135 patients: 57 with iCCA, 43 with pCCA and 35 with dCCA. For 54 patients, corresponding non-neoplastic tissue samples were also examined. Survival data were available for a subset of 116 patients (51 iCCAs, 37 pCCAs and 28 dCCAs).

### 2.2. Tissue Microarray Construction

For generation of tissue microarrays (TMA), 3 μm sections were cut and stained with H&E. Representative areas from the tumor center and non-neoplastic bile duct tissue of the respective region were marked by two experienced pathologists. Tumor tissue and non-neoplastic cores (1.5 mm diameter) were consecutively punched out of the sample tissue blocks and embedded into a new paraffin array block using a tissue microarrayer (Beecher Instruments, Woodland, CA, USA).

### 2.3. Immunohistochemistry

For TIRC7 immunohistochemical staining of CCA TMAs, rabbit polyclonal anti-TIRC7 antibody (CellAct, Dortmund, Germany) was used [20,22]. In brief, 5 µm sections were cut from the formalin-fixed paraffin-embedded samples and deparaffinized and rehydrated according to standard protocols. Sections were then subjected to epitope retrieval at pH8 prior to incubation with anti-TIRC7 antibodies (1:500) for 30 min at room temperature. For detection, the LSAB system was used (Dako REAL Detection System, Agilent Technologies, Santa Clara, CA, USA). For FOXP3 immunohistochemistry, epitope retrieval was performed at pH6. After blocking of endogenous peroxidase (Dako REAL Peroxidase Blocking Solution, Agilent Technologies) sections were incubated with anti-FOXP3 (clone PCH101, 1:50, Thermo Fisher Scientific, Waltham, MA, USA) for 30 min at room temperature followed by incubation with secondary antibody (rabbit anti-rat, 1:1000, Dianova, Hamburg, Germany) for 30 min at room temperature. For detection, EnVision+ Single Reagent (Agilent Technologies, Santa Clara, CA, USA) was used. Nuclei were stained using Mayer’s hemalaun solution (Merck Millipore, Burlington, MA, USA) and sections coverslipped with Kaiser’s glycerol gelatin (Carl Roth GmbH, Karlsruhe, Germany).

### 2.4. Tissue Microarray Evaluation

For each patient, the presence of TIRC7^+^ immune cells was quantified both on tumor and non-neoplastic tissue using TMAs. Only cells with a robust and complete membranous staining were counted. TIRC7-positivity was evaluated both in the epithelial and stromal compartment. In the epithelial compartment, the presence of TIRC7^+^ immune cells was expressed as density of TIRC7^+^ immune cells per epithelial cells. In the stromal tumor compartment, TIRC7-positivity was scored by determination of the absolute number of positive cells present in the stroma on the respective TMA area. Immunohistochemical analysis was always paralleled by verification of cell morphology to exclude false positive or unspecific staining patterns. For statistical analyses, cases were stratified on the basis of the intraepithelial TIRC7^+^ cell density. Since the number of intraepithelial TIRC7^+^ cells in the tumors was low, TIRC7-negative cases were defined as samples with an epithelial TIRC7^+^ immune cell density of 0%, if not otherwise stated, while samples with any positivity (i.e., an intraepithelial density of at least 1%) were defined as being TIRC7-positive. Data on additional immune cell markers were derived from previous studies, as described [15,25]. Accordingly, FOXP3 as the second constituent of the double immunostaining method used was evaluated by counting the absolute number of positive intraepithelial and total cells (per dot). Only cells with a clear nuclear staining were designated FOXP3 positive.

### 2.5. Statistical Analysis

In the case of two group comparisons, differences were assessed using the nonparametric Mann–Whitney U test. In case of three groups, the nonparametric Kruskal–Wallis test was computed followed by Dunn’s test for post hoc pairwise comparisons. Categorical variables were analyzed using the Fisher’s exact or χ2 test, as appropriate. Survival times were graphed using the Kaplan–Meier method and differences assessed by the Mantel–Cox log rank test. The association of two variables was assessed using nonparametric Spearman’s correlation analysis. Statistical analyses were performed with GraphPad Prism 6.0 (GraphPad Software, La Jolla, CA, USA). *p*-values below 0.05 were considered statistically significant.

## 3. Results

### 3.1. TIRC7^+^ Immune Cells Are Present in the Epithelial and Stromal Compartment of Cholangiocarcinoma

**Figure 1:**
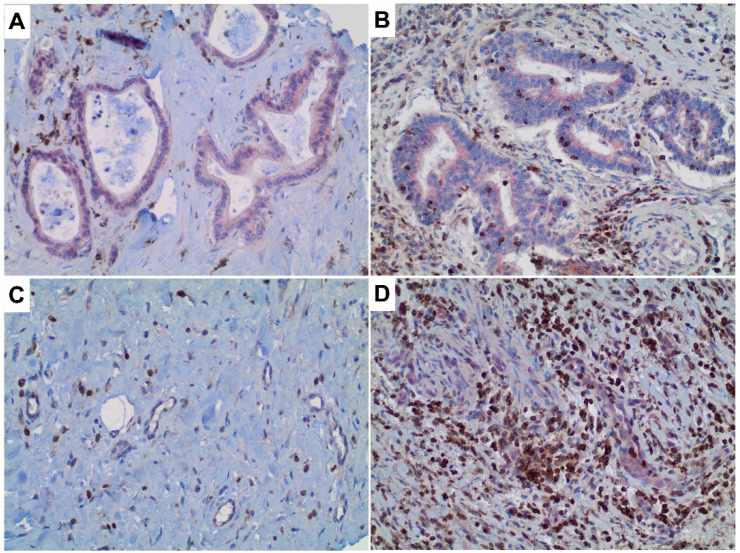
TIRC7^+^ immune cells in cholangiocarcinoma. Photographs of both a low (**A**) and particularly high (**B**) intraepithelial TIRC7^+^ immune cell density and low (**C**) and particularly high (**D**) numbers of TIRC7^+^ cells in the stromal compartment. Displayed are tumor samples. Note that the photographs were used for visualization and as such do not represent the expression average. Original magnification: 200×.

**Figure 2:**
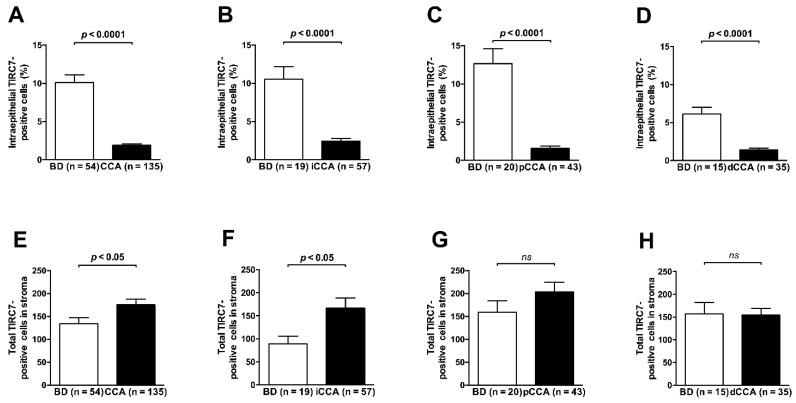
Quantitative analysis of TIRC7^+^ immune cells in tumor and nontumor tissue. Intraepithelial density of TIRC7^+^ immune cells was significantly reduced in tumor tissue as compared to matched non-neoplastic bile duct (BD) epithelia (available for a subset) across all CCAs (**A**) as well as in the subgroup analysis for iCCA (**B**), pCCA (**C**) and dCCA (**D**). Total number of TIRC7^+^ cells in the stromal compartment was significantly higher in tumor as compared to nontumor tissue when all CCAs were included (**E**), which can be attributed to significantly higher levels in iCCA cases (**F**). No significant differences were found regarding pCCA (**G**) and dCCA (**H**) tissue with respect to the stromal compartment. For all analyses the Mann–Whitney U test was used. Data are depicted as mean ± standard error of the mean.

TIRC7^+^ immune cells were found in tumor epithelia in 68.1% of the 135 cases studied, while the stromal compartment harbored TIRC7^+^ immune cells in 134 of 135 samples (99.3%). Intraepithelial density of TIRC7^+^ cells (representative photographs in Figure 1) across all CCA samples was reduced as compared to non-neoplastic bile ducts both in an unpaired (Figure 2A) and paired analysis (each *p* < 0.0001) reaching approximately five times lower mean values (1.9% vs. 10.1%). The difference remained highly statistically significant after stratification for the different anatomical CCA subtypes and separated analysis (Figure 2B–D, *p* < 0.0001 for iCCA, pCCA and dCCA, respectively). Intraepithelial density of TIRC7^+^ cells in non-neoplastic bile ducts did not differ significantly between patients with and without coexisting hepatobiliary disease (median 8% vs. 6%, *p* = 0.46) (Figure S1).

In contrast, the absolute number of TIRC7^+^ immune cells in the stromal compartment was significantly higher in tumoral tissue as compared to non-neoplastic bile ducts (Figure 2E, *p* < 0.05). Subtype-specific analysis revealed a significant stromal difference in the iCCA cohort (Figure 2F, *p* < 0.05) while no difference was observed in pCCA and dCCA (Figure 2G,H). In addition, no correlation was found between intraepithelial TIRC7^+^ density and absolute number of TIRC7^+^ cells in the stromal compartment.

**Figure 3:**
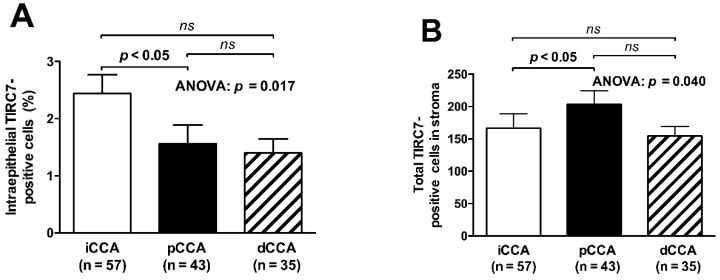
Intergroup analysis of TIRC7^+^ immune cell quantity in tumor tissue. Intraepithelial density of TIRC7^+^ immune cells was significantly different among the three CCA subgroups with significantly higher levels in iCCA compared to pCCA cases (**A**). With respect to the stromal compartment, the total number of TIRC7^+^ cells differed between the groups, being significantly higher for pCCA as compared to iCCA (**B**). Overall group differences were assessed using the Kruskal–Wallis followed by Dunn’s test for post hoc pairwise comparisons. Data are depicted as mean ± standard error of the mean.

Intraepithelial TIRC7^+^ immune cell density varied significantly between the three CCA subgroups (*p =* 0.017) with the highest levels found in iCCA being significantly different from the levels found in pCCA in the post hoc analysis (Figure 3A, *p <* 0.05). Stratification for the different CCA subtypes also revealed significantly different absolute numbers of TIRC7^+^ cells in the stromal compartment (*p =* 0.040), which, in contrast, were attributable to statistically higher levels found in pCCA as compared to iCCA (Figure 3B, *p <* 0.05).

### 3.2. Correlation of TIRC7 Quantity with Clinicopathological Information

After grouping all cases into TIRC7-positive and -negative categories based on the epithelial TIRC7^+^ immune cell density (as defined in the Methods section), characteristics of these two patient groups were analyzed with respect to clinicopathological variables (Table 1). Consistent with the absolute differences observed in tumor intraepithelial TIRC7^+^ immune cell density among the subtypes, contingency analysis showed that TIRC7^+^ and TIRC7 negative cases were not homogeneously distributed by CCA subtype (*p* = 0.004, χ2 test). TIRC7^+^ cases constituted approximately 83% of all iCCA, 66% of all dCCA and 51% of pCCA cases. Considering histopathological subtypes, although statistical analysis for the comparison between conventional (ductal) subtype vs. all other subtypes did not show a significant difference, distinct subtypes displayed an aberrant distribution. All cases exhibiting a signet ring/diffuse (*n* = 7) or adenosquamous morphology (*n* = 2) exclusively belonged to the TIRC7-positive group and were underrepresented in iCCA (*n* = 1) compared to pCCA (*n* = 4) and dCCA (*n* = 4). No differences with respect to age, tumor staging (UICC), tumor grading, infiltration depth, nodal or distant metastasis were found by comparison of the TIRC7-positive and -negative groups. In addition, there was no difference in the presence of concomitant hepatobiliary disease between TIRC7-positive (57/92) and -negative patients (28/43) (*p* = 0.85).

To examine the influence of threshold selection, we repeated the analyses using the 85th percentile of each measure as cut-off values for both the intraepithelial and stromal compartment, respectively. Interestingly, using such a high threshold for intraepithelial TIRC7+ immune cell density revealed a significant difference in UICC stage with TIRC7-positive patients presenting at a later disease stage compared to TIRC7-negative patients (Table S1, *p* = 0.047). No other correlations were found with respect to the epithelial or stromal compartments (Table S2).

### 3.3. High Intraepithelial TIRC7^+^ Immune Cell Density Is Associated with Favorable Outcome in Intrahepatic Cholangiocarcinoma

**Figure 4:**
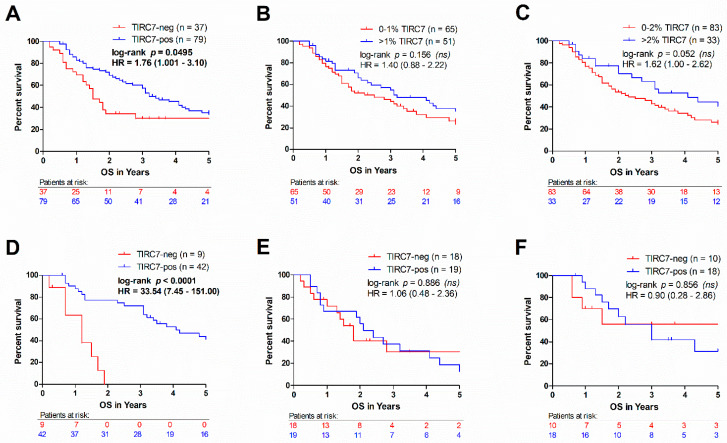
Survival analysis in relation to TIRC7 quantity. Cases with absent TIRC7^+^ intraepithelial cells showed a remarkably shortened survival as compared to TIRC7-positive cases across all CCA types (**A**). An analogous trend was also seen when using the median (**B**) and the mean (**C**) value of intraepithelial TIRC7^+^ immune cell density as stratification cut-offs. Subgroup analysis revealed a strong survival benefit of TIRC7-positive cases in iCCA (**D**), while no differences were found with respect to pCCA (**E**) and dCCA (**F**). *p*-values were computed using log-rank testing. Hazard ratios (HR) and its 95% confidence intervals were calculated using the Mantel Haenszel approach.

As immune cell infiltration has been reported to influence patient outcome, we examined the relationship of TIRC7^+^ immune cells to overall survival in CCA. Overall survival information was available for 116 patients, of whom 68% were TIRC7-positive. We found that the absolute number of TIRC7^+^ cells in the stromal compartment was not associated with overall survival in the whole population nor in any of the subtypes irrespective of the used cut-off value. Next, we assessed the impact of intraepithelial TIRC7^+^ immune cell density on patient prognosis and compared the overall survival of CCA patients with or without intraepithelial TIRC7^+^ immune cell infiltration. CCA patients with absent intraepithelial TIRC7^+^ immune cells showed a significantly shortened survival as compared to all other cases (Figure 4A, *p =* 0.0495). In fact, the median survival in the TIRC7-negative group was more than halved as compared to the TIRC7-positive cohort (1.5 vs. 3.2 years, hazard ratio = 1.76). A trend towards better survival in TIRC7-positive patients was also observed when the median (Figure 4B, *p =* 0.156, median = 1%) or the mean (Figure 4C, *p <* 0.052, mean = 2%) were used as the cut-off for TIRC7 positivity, supporting the association between intraepithelial TIRC7^+^ immune cell quantity and overall survival. Subtype-specific analysis revealed that the observed findings were largely attributable to differences in iCCA survival (Figure 4D, *p <* 0.0001, medial survival 1.2 vs. 4.1 years, hazard ratio = 33.5). No meaningful differences were observed for pCCA or dCCA cases (Figure 4E,F). This observation was also made when the 85th percentile of intraepithelial TIRC7^+^ immune cell density was used as the threshold, revealing a significantly decreased survival of the TIRC7-negative group in iCCA (Figure S2B, *p =* 0.008, hazard ratio = 2.81) but not the other subtypes (Figure S2C,D).

### 3.4. Intraepithelial TIRC7^+^ Immune Cell Density in Cholangiocarcinoma Is Associated with the Number of Intraepithelial CD8^+^ and Total CD4^+^ Lymphocytes

**Figure 5:**
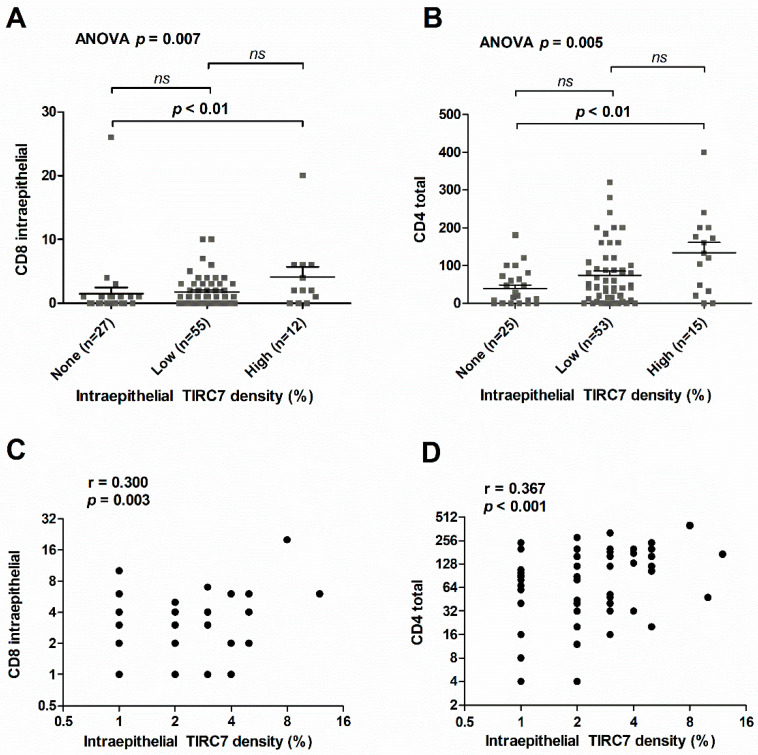
Association of intraepithelial TIRC7^+^ immune cell density with immune cell phenotype. Analysis of quantity of intraepithelial CD8^+^ (**A**) or total CD4^+^ (**B**) T cells in CCA cases with absent (0%), low (1–3%) or high (≥4%) intraepithelial TIRC7^+^ immune cell density shown by scatter dot plots with mean and SEM (CD8 Kruskal–Wallis *p =* 0.007; CD4 Kruskal–Wallis *p =* 0.005). Correlation of intraepithelial CD8^+^ (**C**) or total CD4^+^ (**D**) T cells with intraepithelial TIRC7^+^ immune cell density, as indicated (CD8 Spearman r = 0.300, *p =* 0.003; CD4 Spearman r = 0.367, *p <* 0.001). Overall group comparison was performed using the Kruskal–Wallis followed by Dunn’s test for post hoc pairwise comparisons (**A**,**B**). Correlation metrics were computed out using Spearman’s correlation analysis (**C**,**D**).

To understand the association between TIRC7^+^ immune cells and the composition of the immune infiltrate, we investigated the correlation of intraepithelial TIRC7^+^ immune cell density with the presence of CD4^+^, CD8^+^, CD20^+^, CD25^+^, CD68^+^ and FOXP3^+^ immune cells assessed in a previous study (Table 2) [15]. Among those, an increasing total CD4^+^ and intraepithelial CD8^+^ immune cell infiltrate was seen towards higher levels of TIRC7^+^ immune cell density by stratification for cases with absent (0%), intermediate (1–3%) and high (≥4%) TIRC7^+^ immune cell quantity (Figure 5A,B, CD8^+^ Kruskal–Wallis *p =* 0.007; CD4^+^ Kruskal–Wallis *p =* 0.005). Post hoc analysis confirmed a statistically significant difference between cases with absent TIRC7^+^ immune cells and a high TIRC7^+^ immune cell density (Figure 5A,B, *p <* 0.01 for CD4^+^ and CD8^+^, respectively). Substantiating these findings, correlation analysis demonstrated a significant correlation between individual intraepithelial TIRC7^+^ immune cell density and the presence of total CD4^+^ and intraepithelial CD8^+^ T lymphocytes, respectively (Figure 5C,D and Table 2, CD8^+^ Spearman r = 0.300, *p =* 0.003; CD4^+^ Spearman r = 0.367, *p <* 0.001). In addition, a trend towards higher levels of total number CD20^+^ immune cells was observed with increasing TIRC7 quantity (r = 0.296, *p* = 0.051). Since TIRC7 was part of a double immunostaining process along with FOXP3 expressed by regulatory T cells, we also assessed intraepithelial and total FOXP3 quantity in CCA. However, in the vast majority of cases, FOXP3 was absent in the tumor epithelium and no correlations with TIRC7^+^ immune cell density were observed both with respect to intraepithelial or total FOXP3 immune cell count (Table 2). After adjustment for multiple testing, no significant correlations of intraepithelial TIRC7^+^ density with immune cell markers specific to macrophages (CD68) or regulatory T cell subsets (CD25 and FOXP3) were identified. In summary, we found that the density of TIRC7^+^ immune cells in the tumor epithelia of individual CCA patients is markedly reduced compared to non-tumorous bile ducts and variable between the different anatomic subtypes. The absence of intraepithelial TIRC7^+^ immune cells was identified as a significant negative prognosticator in iCCA.

## 4. Discussion

As the tumor microenvironment plays a crucial role in cancer progression and targeting the immune system has become a promising therapeutic opportunity, it is important to better understand the composition and role of distinct immune cell populations within the tumor microenvironment. This study was built on these breakthrough advancements in the field of oncology and aimed to examine the presence of a distinct subset of immune cells expressing the transmembrane protein TIRC7 in CCA and to analyze its correlation with clinicopathological parameters. As a pilot study, our results provide a foundation for future studies exploring the prospects of its pharmacological modulation.

TIRC7 exerts immunoinhibitory properties and most studies point towards an inhibitory role with respect to lymphocyte activation. In TIRC7-deficient mice created by gene targeting, TIRC7 deficiency led to increased proliferation and enhanced activation of T cells along with an augmented hypersensitivity response [18]. Potentially therapeutic agonist-TIRC7-antibodies not only ameliorated both collagen-induced arthritis and increased survival of allografts in rodents, but also exhibited inhibitory effects on ex vivo proliferation of lymphocytes obtained from patients suffering from multiple sclerosis [20,21,22,23]. Despite these promising findings on a potential immunoinhibitory role of TIRC7, the evidence for a role of TIRC7 in cancer development or progression is limited to a few recent studies on selected tumor entities. Meng et al. have identified an aberrant splicing product of TIRC7 pre-mRNA to be associated with worse survival and metastasis in silico in renal clear cell carcinoma [26]. In glioblastoma multiforme, increased expression of TIRC7 was shown to be associated with both the immune cell composition and prognosis [27]. However, it needs to be noted that in both studies TIRC7 expression was assessed in the tumor itself instead of analyzing the implications of infiltrating TIRC7^+^ immune cells.

Here, we showed in CCA patients that TIRC7^+^ immune cells are present in the stroma of over 99% and in the epithelium of 68% of cases, while the presence of TIRC7^+^ immune cells in the tumor epithelia of CCA patients is markedly reduced compared to non-tumorous bile ducts. Though the presence of coexisting hepatobiliary diseases in the majority of patients and cholestasis phenomena may have contributed to the comparably high number of TIRC7^+^ immune cells in non-neoplastic bile ducts, the remarkable difference to CCA tissue indicates a state of increased immunorecognition as part of the host antitumor immune response. In turn, the number of TIRC7^+^ immune cells in the surrounding stroma was increased compared to non-tumorous tissue, possibly mediated by tumor-induced stromal factors promoting immune evasion. It may be speculated that the high intraepithelial TIRC7 levels found in iCCA may be at least partially attributed to the particularly dense vascularization of the liver. Indeed, using single-cell RNA sequencing, recently Fabris et al. demonstrated that iCCAs harbor an exceptionally complex and diverse ecosystem that is integral to tumor progression [28].

Correlation of TIRC7^+^ immune cell quantity with clinicopathological information suggests that distinct morphological subtypes, such as signet ring cell or adenosquamous carcinoma, seem to be particularly prone to epithelial infiltration by TIRC7^+^ immune cells. Though the generalizability of this finding is strongly limited given the small number of these histotypes included in this study, the clear-cut separation by TIRC7 expression and the concordance with previous studies on other immune exhaustion markers may point towards an optimized capability of these tumors to evade the immune system by induction of TIRC7. In a recent study of PD-L1 expression in gallbladder cancer, the same histological subtypes also demonstrated the highest levels of PD-L1 expression among the whole cohort, underpinning a particularly strong impact of these tumors on the local immune response [29]. In accordance, a previous study demonstrated that these histological subtypes were also much more likely to exhibit microsatellite instability, which is closely associated with a high mutational burden and subsequently enhanced expression of immune checkpoints [30]. Since we found these particular histopathological subtypes to be underrepresented in iCCA compared to pCCA and dCCA, the finding of particularly high TIRC7 levels in iCCA cannot be attributed to a heterogeneous distribution of histopathological subtypes.

Analysis of overall survival showed that the presence of intraepithelial TIRC7^+^ immune cells seems to be protective as TIRC7 positivity was accompanied by a more than doubled median survival compared to patients without intraepithelial TIRC7^+^ immune cell infiltrate. Subtype-specific testing revealed that this effect was attributable to a large and highly significant survival difference in iCCA patients, whereas for both pCCA and dCCA survival curves did not differ between the TIRC7-positive and -negative groups. This finding may seem counter-intuitive given the immunoinhibitory properties of TIRC7. However, as the expression of most immunoinhibitory factors is largely dependent on specific cytokines, such as interferon-γ in the context of PD-L1, abundance of TIRC7^+^ immune cells may rather be surrogative of a pronounced local anti-tumor response rather than a state of effective immune evasion [31]. In keeping with this, high PD-L1 expression was shown to be a predictor of improved survival in several studies across different solid tumor entities, e.g., gastric cancer, breast cancer and malignant melanoma [32,33,34]. This perception is substantiated by the strong association of intraepithelial TIRC7 density with the number of cytotoxic T cells detected in this study. The fact that a signification association was only found with respect to the intraepithelial but not stromal TIRC7^+^ immune cell quantity may further support this view, as epithelial infiltration by immune cells is mostly observed in settings of advanced local inflammation. Due to the inhibitory properties of TIRC7 on T cell function and other immune parameters, with respect to the PD-1/PD-L1 axis, potential drugs should be designed to abrogate its function in order to unleash the immune system, e.g., by using antagonistic TIRC7-directed antibodies.

As for immunohistochemical markers, threshold selection to stratify subjects into negative and positive groups is critical. For TIRC7, however, given its novelty there is no consensus cut-off value. In this study, only the complete absence of TIRC7^+^ immune cells was defined as TIRC7-negative, whereas any expression was rendered positive. This definition was made empirically based on the overall low number of TIRC7^+^ immune cells. It follows the assumption that misclassification of a positive sample to the negative group outweighs the danger of vice versa. Moreover, choosing a cut-off value within the relatively low range of positive values is much more prone to observer variability than defining any expression as positive in a clear-cut manner. Last but not least, we adhered to the majority of studies on PD-L1 which stipulated the same value (1%) as the decision threshold across a variety of solid tumor entities [35,36]. The fact that the remarkable difference in prognosis between both groups in iCCA was less pronounced but persisted when using the 85th percentile as a cut-off value underscores the prognostic significance of intraepithelial TIRC7^+^ immune cell density and justifies our approach of threshold determination.

By correlating our results with immune cell infiltrate characteristics in the same cohort, we found that high intraepithelial TIRC7^+^ immune cell densities were paralleled by an increased number of both intraepithelial CD8^+^ cytotoxic and total number of CD4^+^ T cells. The association with intraepithelial CD8^+^ cells may emphasize that TIRC7 is not only expressed by CD4^+^, but also cytotoxic CD8^+^ T cells, as observed in a collagen-induced arthritis mouse model [20].

Though there is a fast-growing body of research on immune oncology, among the studied immune checkpoints, the PD-1/PD-L1 and CTLA-4 axes stand out by successful transition from the laboratory to the clinic. Both pathways interfere with the activation of T cells, which in addition to binding of the T cell receptor to MHC, require engagement of CD28 on the antigen-presenting cell as a second, costimulatory signal. CTLA-4 is a homolog of CD28, that however does not provide a stimulatory signal upon binding to B7 and as such acts as a competitive inhibitor to T cell activation [37]. Interestingly, there is accumulating evidence that TIRC7 may exert its effects, at least partially, by modulation of CTLA-4. In 2006, we demonstrated that TIRC7 colocalizes with CTLA-4 in human T cells and showed that the TIRC7-induced inhibitory effects on T cells are dependent on CTLA-4 expression, as a blockade of CTLA-4 completely abolished TIRC7-mediated effects [38]. A similar finding was reported on lymphocytes from patients with acute graft-versus-host disease by Zhu et al., who concluded that TIRC7 inhibits T cell activation by upregulation of CTLA-4 [39]. Given the breakthrough advancements achieved by inhibition of CTLA-4 in several advanced tumor entities and the evidence on its functional relation to TIRC7, TIRC7 should be considered a potential new upstream molecule to be addressed therapeutically in CCA and other solid tumors.

Major limitations of this study are its retrospective design and the concomitant absence of direct functional data on the relevance of TIRC7. As such, this study objectifies the presence of TIRC7^+^ immune cells in CCA, but by nature cannot establish its potential as a therapeutic target. It provides a solid database for further studies exploring the implications of TIRC7 modulation in CCA and other solid tumors.

## 5. Conclusions

In conclusion, we described the presence of TIRC7^+^ immune cells in the epithelial and stromal compartment of CCA and showed that the absence of intraepithelial TIRC7^+^ cells is strongly associated with poor overall survival in iCCA patients. This study objectified the presence of TIRC7^+^ immune cells in all subtypes of CCA and demonstrated their intraepithelial quantity to be a significant positive prognosticator in iCCA. Given the immunoinhibitory effects of TIRC7 and its interaction with CTLA-4, future studies should explore the impact of TIRC7-modulation alone or in combination with immune therapy in CCA and other malignancies.

## Figures and Tables

**Table 1:** Clinicopathological characteristics stratified for intraepithelial TIRC7-positivity.

Clinicopathological Variable		Total 135 (100.0)	TIRC7 Negative 43 (31.9)	TIRC7 Positive 92 (68.1)	*p*-Value ^1^
Age	Median	63.0	64.10	63.5	0.692
	Mean	61.7	61.7	62.3	
	Interquartile Range	55.0–69.0	56.0–69.4	55.2–70.2	
Sex	Male	89 (100.0)	32 (36.0)	57 (64.0)	0.177
	Female	46 (100.0)	11 (23.9)	35 (76.1)	
Subtype	iCCA	57 (100.0)	10 (17.5)	47 (82.5)	**0.004**
	pCCA	43 (100.0)	21 (48.9)	22 (51.1)	
	dCCA	35 (100.0)	12 (34.3)	23 (65.7)	
Histology ^2^	Ductal	89 (100.0)	30 (33.7)	59 (66.3)	0.520 ^3^
	Papillary	9 (100.0)	3 (33.3)	6 (66.7)	
	Mucinous	5 (100.0)	3 (60.0)	2 (40.0)	
	Solid	13 (100.0)	3 (23.1)	10 (76.9)	
	Diffuse/signet ring	7 (100.0)	0 (0.0)	7 (100.0)	
	Intestinal	2 (100.0)	2 (100.)	0 (0.0)	
	Adenosquamous	2 (100.0)	0 (0.0)	2 (100.0)	
	Clear cell	8 (100.0)	2 (25.0)	6 (75.0)	
UICC ^4^	UICC 1	6 (100.0)	3 (50.0)	3 (50.0)	0.915
	UICC 2	51 (100.0)	18 (35.3)	33 (64.7)	
	UICC 3	35 (100.0)	13 (37.1)	22 (62.9)	
	UICC 4	5 (100.0)	2 (40.0)	3 (60.0)	
	NA	38 (100.0)	7 (18.4)	31 (81.6)	
pT	T1	10 (100.0)	3 (30.0)	7 (70.0)	0.955
	T2	77 (100.0)	26 (33.8)	51 (66.2)	
	T3	37 (100.0)	11 (29.7)	26 (70.3)	
	T4	11 (100.0)	3 (27.3)	8 (72.7)	
pN	N0	46 (100.0)	16 (34.8)	30 (65.2)	0.706
	N1	52 (100.0)	20 (38.5)	32 (61.5)	
	NA	37 (100.0)	7 (18.9)	30 (81.1)	
M	M0	131 (100.0)	41 (31.3)	90 (68.7)	0.592
	M1	4 (100.0)	2 (50.0)	2 (50.0)	
G	G1	8 (100.0)	3 (37.5)	5 (62.5)	0.875
	G2	99 (100.0)	32 (32.3)	67 (67.7)	
	G3	28 (100.0)	8 (28.6)	20 (71.4)	
R	R0	65 (100.0)	20 (30.8)	45 (69.2)	0.958
	R1	44 (100.0)	14 (31.8)	30 (68.2)	
	R2	11 (100.0)	3 (27.3)	8 (72.7)	
	NA	15 (100.0)	6 (40.0)	9 (60.0)	
L/V	L/V0	34 (100.0)	11 (32.4)	23 (67.6)	1.000
	L/V1	101 (100.0)	32 (31.7)	69 (68.3)	
Pn	Pn0	65 (100.0	16 (24.6)	49 (75.4)	0.098
	Pn1	70 (100.0)	27 (38.6)	43 (61.4)	
Hepatobiliary	HBV	11 (100.0)	4 (36.4)	7 (63.6)	0.345
Disease ^5^	HCV	2 (100.0)	0 (0.0)	2 (100.0)	
	Cholecystitis/-lithiasis	43 (100.0)	10 (23.3)	33 (76.7)	
	High-stage fibrosis/cirrhosis	31 (100.0)	11 (35.5)	20 (64.5)	
	Fatty liver disease	13 (100.0)	3 (23.1)	10 (76.9)	
	PSC	2 (100.0)	2 (100.0)	0 (0.0)	
	Chronic pancreatitis	3 (100.0)	1 (33.3)	2 (66.7)	
	Hemochromatosis	2 (100.0)	1 (50.0)	1 (50.0)	
	Siderosis	5 (100.0)	0 (0.0)	5 (100.0)	
	None identified	50 (100.0)	15 (30.0)	35 (70.0)	
Overall survival	Median survival in years (*n*)	3.0 (116)	1.5 (37)	3.2 (79)	

Unless otherwise noted, data are depicted as absolute numbers (%). ^1^ *p*-values were calculated using Fisher’s Exact test or χ²-test as appropriate excluding missing data (NA), bold *p*-values indicate significant values; ^2^ For tumors with mixed histological type the predominant histological phenotype other than ductal was denoted; ^3^ Comparison was carried out for the groups ductal vs. all other subtypes (pooled); ^4^ Cases with pNx had no lymph nodes resected, therefore, UICC status could not be assessed; ^5^ Patients with multiple diseases are counted in each category. Abbreviations: iCCA, Intrahepatic cholangiocarcinoma; pCCA, perihilar cholangiocarcinoma; dCCA, extrahepatic cholangiocarcinoma; UICC 1–4, Union for International Cancer Control stages 1–4; pT, histopathologic tumor stage evaluation; pN, histopathologic lymph node evaluation; M, distant metastases; G, grade of differentiation; R, resection margins; L/V, invasion into lymphatic vessels or veins; Pn, perineural invasion; HBV, hepatitis B virus; HCV, hepatitis C virus; PSC, primary sclerosing cholangitis.

**Table 2:** Association of intraepithelial TIRC7+ immune cell density with immune cell composition.

Immune Cell Metric	Intraepithelial TIRC7 Density		
	*r* Value	*p*-Value ^1^	adj. *p*-Value ^2^
CD4 intraepithelial	0.164	0.116	1.000
CD4 total	0.367	**<0.001**	**0.004**
CD8 intraepithelial	0.300	**0.003**	**0.043**
CD8 total	0.083	0.427	1.000
CD20 intraepithelial	NA	NA	NA
CD20 total	0.296	0.004	0.051
CD68 intraepithelial	0.013	0.902	1.000
CD68 total	−0.033	0.748	1.000
CD25 intraepithelial	0.037	0.721	1.000
CD25 total	0.190	0.066	0.861
FoxP3 intraepithelial	0.184	0.080	1.000
FoxP3 total	0.150	0.156	1.000

Displayed are the metrics for correlations between intraepithelial TIRC7^+^ immune cell density and the quantity of immune cell subsets expressing specific markers in either the intraepithelial or stromal compartment. ^1^ *p*-values were calculated using Spearman’s correlation analysis. ^2^ *p*-values were adjusted by Bonferroni correction. Bold *p*-values indicate significant values.

## Data Availability

Additional datasets analyzed during the current study are available from the corresponding author upon reasonable request.

## Supplementary Materials

The following are available online at https://www.mdpi.com/article/10.3390/cancers13246272/s1, Figure S1: Intraepithelial TIRC7^+^ immune cell density in non-neoplastic bile ducts stratified for the presence of hepatobiliary disease. Intraepithelial TIRC7 immune cell density in non-neoplastic bile ducts did not differ significantly between patients with (*n* = 34) and without (*n* = 20) concomitant hepatobiliary disease. For statistical comparison, Mann-Whitney U test was used. Data are depicted as mean ± standard error of the mean, Figure S2: Survival analysis using the 85th percentile of intraepithelial TIRC7^+^ immune cell density as cut-off value. While no significant differences were found with respect to CCAs in general (**A**), subgroup analysis revealed a significantly improved survival of the TIRC7^-^ positive group in iCCA patients (**B**). No significant differences between both groups were found with respect to pCCA (**C**) and dCCA (**D**). *p*-values were computed using log-rank testing. Hazard ratios (HR) and its 95% confidence intervals were calculated using the Mantel Haenszel approach, Table S1: Cut-off 85th percentile for intraepithelial TIRC7^+^ immune cell density, Table S2. Cut-off 85th percentile for stromal TIRC7 quantity.
