# Supplementary material for: The Transmembrane Receptor TIRC7 Identifies a Distinct Subset of Immune Cells with Prognostic Implications in Cholangiocarcinoma
Source: Cancers (Basel). 2021 Dec 14;13(24):6272. doi: 10.3390/cancers13246272 (PMC8699724; doi:10.3390/cancers13246272)
Supplement: Supplementary file 1 [file cancers-13-06272-s001.zip › cancers-1503765-supplementary.pdf]

Supplementary materials: The Transmembrane Receptor TIRC7 Identifies a Distinct Subset of Immune Cells with Prognostic Implications in Cholangiocarcinoma

Thomas Albrecht, Benjamin Goeppert, Fritz Brinkmann, Alphonse Charbel, Qiangnu Zhang, Johannes Schreck, Nina Wilhelm, Stephan Singer, Bruno C. Köhler, Christoph Springfeld, Arianeb Mehrabi, Peter Schirmacher, Anja A. Kühl, Monika N. Vogel, Holger Jansen, Nalân Utku and Stephanie Roessler

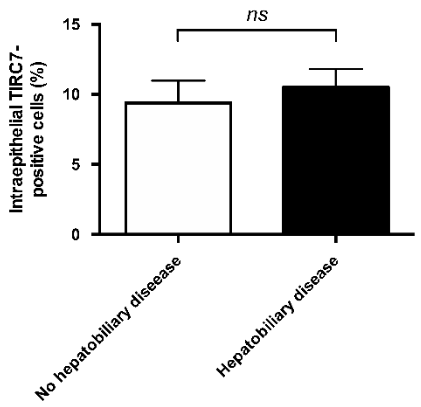

Figure S1. Intraepithelial TIRC7⁺ immune cell density in non-neoplastic bile ducts stratified for the presence of hepatobiliary disease. Intraepithelial TIRC7 immune cell density in non-neoplastic bile ducts did not differ significantly between patients with (n = 34) and without (n = 20) concomitant hepatobiliary disease. For statistical comparison, Mann-Whitney U test was used. Data are depicted as mean ± standard error of the mean.

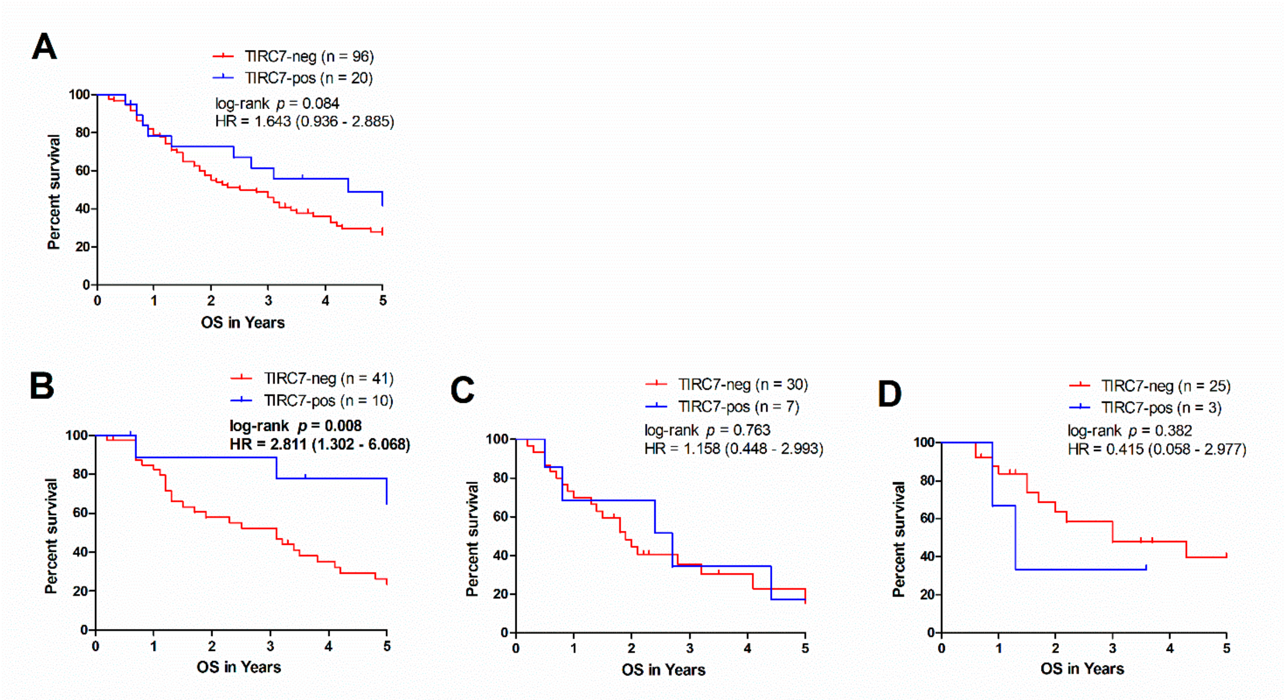

Figure S2. Survival analysis using the 85th percentile of intraepithelial TIRC7⁺ immune cell density as cut-off value. While no significant differences were found with respect to CCAs in general (**A**), subgroup analysis revealed a significantly

improved survival of the TIRC7- positive group in iCCA patients (**B**). No significant differences between both groups were found with respect to pCCA (**C**) and dCCA (**D**). *P*-values were computed using log-rank testing. Hazard ratios (HR) and its 95% confidence intervals were calculated using the Mantel Haenszel approach.

Table S1. Cut-off 85th percentile for intraepithelial TIRC7+ immune cell density.

		**Total**	**TIRC7 Negative**	**TIRC7 Positive**	***p*-Value**[1]
All patients		135 (100.0)	113 (83.7)	22 (16.3)	
Age	Median	63.0	63.3	65.2	0.617
	Mean	61.7	61.9	63.0	
	Interquartile Range	55.0–69.0	54.7–70.0	55.8-69.5	
Sex	Male	89 (100.0)	73 (82.0)	16 (18.0)	0.624
	Female	46 (100.0)	40 (86.7)	6 (13.3)	
Subtype	iCCA	57 (100.0)	45 (78.9)	12 (21.1)	0.290
	pCCA	43 (100.0)	36 (83.7)	7 (16.3)	
	dCCA	35 (100.0)	32 (91.4)	3 (8.6)	
Histology[2]	Ductal	89 (100.0)	76 (85.4)	13 (14.6)	0.470[3]
	Papillary	9 (100.0)	7 (77.8)	2 (22.2)	
	Mucinous	5 (100.0)	5 (100.0)	0 (0.0)	
	Solid	13 (100.0)	9 (69.2)	4 (30.8)	
	Diffuse/signet ring	7 (100.0)	5 (71.4)	2 (28.6)	
	Intestinal	2 (100.0)	2 (100.0)	0 (0.0)	
	Adenosquamous	2 (100.0)	2 (100.0)	0 (100.0)	
	Clear cell	8 (100.0)	7 (87.5)	1 (12.5)	
UICC[4]	UICC 1	6 (100.0)	6 (100.0)	0 (0.0)	**0.047**[5]
	UICC 2	51 (100.0)	48 (94.1)	3 (5.9)	
	UICC 3	35 (100.0)	29 (82.9)	6 (17.1)	
	UICC 4	5 (100.0)	3 (60.0)	2 (40.0)	
	NA	38 (100.0)	27 (71.1)	11 (28.9)	
pT	T1	10 (100.0)	7 (70.0)	3 (30.0)	0.469[6]
	T2	77 (100.0)	64 (83.1)	13 (16.9)	
	T3	37 (100.0)	32 (86.5)	5 (13.5)	
	T4	11 (100.0)	10 (90.9)	1 (9.1)	
pN	N0	46 (100.0)	39 (84.8)	7 (15.2)	0.540
	N1	52 (100.0)	47 (90.4)	5 (9.6)	
	NA	37 (100.0)	27 (73.0)	10 (27.0)	
M	M0	131 (100.0)	110 (84.0)	21 (16.0)	0.513
	M1	4 (100.0)	3 (75.0)	1 (25.0)	
G	G1	8 (100.0)	6 (75.0)	2 (25.0)	0.566
	G2	99 (100.0)	82 (82.8)	17 (17.2)	
	G3	28 (100.0)	25 (89.3)	3 (10.7)	
R	R0	65 (100.0)	52 (80.0)	13 (20.0)	0.340
	R1	44 (100.0)	39 (88.6)	5 (11.4)	
	R2	11 (100.0)	8 (72.7)	3 (27.3)	
	NA	15 (100.0)	14 (93.3)	1 (6.7)	
L/V	L/V0	34 (100.0)	28 (82.4)	6 (17.6)	0.793
	L/V1	101 (100.0)	85 (84.2)	16 (15.8)	
Pn	Pn0	65 (100.0	52 (80.0)	13 (20.0)	0.352
	Pn1	70 (100.0)	61 (87.1)	9 (12.9)	
Hepatobiliary Disease[7]	HBV	11 (100.0)	10 (90.9)	1 (9.1)	0.825[8]
	HCV	2 (100.0)	1 (50.0)	1 (50.0)	
	Cholecystitis/-lithiasis	43 (100.0)	36 (83.7)	7 (16.3)	
	High-stage fibrosis/cirrhosis	31 (100.0)	27 (87.1)	4 (12.9)	
	Fatty liver disease	13 (100.0)	9 (69.2)	4 (30.8)	
	PSC	2 (100.0)	2 (100.0)	0 (0.0)	
	Chronic pancreatitis	3 (100.0)	2 (66.7)	1 (33.3)	

	Hemochromatosis	2 (100.0)	2 (100.0)	0 (0.0)
	Siderosis	5 (100.0)	2 (40.0)	3 (60.0)
	None identified	50 (100.0)	42 (84.0)	8 (16.0)
Overall survival	Median survival in years (*n*)	3.0 (116)	2.8 (96)	4.4 (20)

Unless otherwise noted, data are depicted as absolute numbers (%). [1] *p*-values were calculated using Fisher's Exact test or χ^2-test as appropriate excluding missing data (NA), bold *p*-values indicate significant values; [2] For tumors with mixed histological type the predominant histological phenotype other than ductal was denoted; [3] Comparison was carried out for the groups ductal *vs.* all other subtypes (pooled); [4] Cases with pNx had no lymph nodes resected, therefore, UICC status could not be assessed; [5] Comparison was carried out for the groups UICC1+2 vs. UICC3+4; [6] Comparison was carried out for the groups pT1+2 vs. pt3+4; [7] Patients with multiple diseases are counted in each category; [8] Comparison was carried out for the groups any vs none. Abbreviations: iCCA, Intrahepatic cholangiocarcinoma; pCCA, Perihilar cholangiocarcinoma; dCCA, Extrahepatic cholangiocarcinoma; UICC 1–4, Union for International Cancer Control stages 1–4; pT, Histopathologic tumor stage evaluation; pN, Histopathologic lymph node evaluation; M, Distant metastases; G, Grade of differentiation; R, Resection margins; L/V, Invasion into lymphatic vessels or veins; Pn, Perineural invasion; HBV, Hepatitis B virus; HCV, Hepatitis C virus; PSC, Primary sclerosing cholangitis.

Table S2. Cut-off 85th percentile for stromal TIRC7 quantity.

		Total	TIRC7 Negative	TIRC7 Positive	*p*-Value[1]
All patients		135 (100.0)	116 (85.9)	19 (14.1)	
Age	Median	63.0	63.1	65.3	0.246
	Mean	61.7	61.6	65.4	
	Interquartile Range	55.0–69.0	54.1–69.8	58.6–73.4	
Sex	Male	89 (100.0)	78 (87.6)	11 (12.4)	0.443
	Female	46 (100.0)	38 (82.6)	8 (17.4)	
Subtype	iCCA	57 (100.0)	49 (86.0)	8 (14.0)	0.829
	pCCA	43 (100.0)	36 (83.7)	7 (16.3)	
	dCCA	35 (100.0)	31 (88.6)	4 (11.4)	
Histology[2]	Ductal	89 (100.0)	73 (82.0)	16 (18.0)	0.115[3]
	Papillary	9 (100.0)	9 (100.0)	0 (0.0)	
	Mucinous	5 (100.0)	5 (100.0)	0 (0.0)	
	Solid	13 (100.0)	12 (92.3)	1 (7.7)	
	Diffuse/signet ring	7 (100.0)	6 (85.7)	1 (14.3)	
	Intestinal	2 (100.0)	2 (100.0)	0 (0.0)	
	Adenosquamous	2 (100.0)	2 (100.0)	0 (0.0)	
	Clear cell	8 (100.0)	7 (87.5)	1 (12.5)	
UICC[4]	UICC 1	6 (100.0)	6 (100.0)	0 (0.0)	0.394[5]
	UICC 2	51 (100.0)	44 (86.3)	7 (13.7)	
	UICC 3	35 (100.0)	29 (82.9)	6 (17.1)	
	UICC 4	5 (100.0)	3 (60.0)	2 (40.0)	
	NA	38 (100.0)	34 (89.5)	4 (10.5)	
pT	T1	10 (100.0)	7 (70.0)	3 (30.0)	0.607[6]
	T2	77 (100.0)	69 (89.6)	8 (10.4)	
	T3	37 (100.0)	32 (86.5)	5 (13.5)	
	T4	11 (100.0)	8 (72.7)	3 (27.3)	
pN	N0	46 (100.0)	40 (87.0)	6 (13.0)	0.585
	N1	52 (100.0)	42 (80.8)	10 (19.2)	
	NA	37 (100.0)	34 (91.9)	3 (8.1)	
M	M0	131 (100.0)	113 (86.3)	18 (13.7)	0.459
	M1	4 (100.0)	3 (75.0)	1 (25.0)	
G	G1	8 (100.0)	6 (75.0)	2 (25.0)	0.591
	G2	99 (100.0)	85 (85.9)	14 (14.1)	
	G3	28 (100.0)	25 (89.3)	3 (10.7)	
R	R0	65 (100.0)	55 (84.6)	10 (15.4)	0.851
	R1	44 (100.0)	38 (86.4)	6 (13.6)	
	R2	11 (100.0)	10 (90.9)	1 (9.1)	
	NA	15 (100.0)	13 (86.7)	2 (13.3)	
L/V	L/V0	34 (100.0)	29 (85.3)	5 (14.7)	1.000

	L/V1	101 (100.0)	87 (86.1)	14 (13.9)	
Pn	Pn0	65 (100.0	57 (87.7)	8 (12.3)	0.627
	Pn1	70 (100.0)	59 (84.3)	11 (15.7)	
Hepatobiliary Disease[7]	HBV	11 (100.0)	9 (81.8)	2 (18.2)	0.808[8]
	HCV	2 (100.0)	1 (50.0)	1 (50.0)	
	Cholecystitis/-lithiasis	43 (100.0)	40 (93.0)	3 (7.0)	
	High-stage fibrosis/cirrhosis	31 (100.0)	27 (87.1)	4 (22.9)	
	Fatty liver disease	13 (100.0)	10 (76.9)	3 (23.1)	
	PSC	2 (100.0)	2 (100.0)	0 (0.0)	
	Chronic pancreatitis	3 (100.0)	2 (66.7)	1 (33.3)	
	Hemochromatosis	2 (100.0)	1 (50.0)	1 (50.0)	
	Siderosis	5 (100.0)	3 (60.0)	2 (40.0)	
	None identified	50 (100.0)	44 (88.0)	6 (12.0)	
Overall survival	Median survival in years (*n*)	3.0 (116)	3.0 (101)	2.7 (15)	

Unless otherwise noted, data are depicted as absolute numbers (%). [1] *p*-values were calculated using Fisher's Exact test or χ^2-test as appropriate excluding missing data (NA), bold *p*-values indicate significant values; [2] For tumors with mixed histological type the predominant histological phenotype other than ductal was denoted; [3] Comparison was carried out for the groups ductal *vs.* all other subtypes (pooled); [4] Cases with pNx had no lymph nodes resected, therefore, UICC status could not be assessed; [5] Comparison was carried out for the groups UICC1+2 vs. UICC3+4; [6] Comparison was carried out for the groups pT1+2 vs. pt3+4; [7] Patients with multiple diseases are counted in each category; [8] Comparison was carried out for the groups any vs none. Abbreviations: iCCA, Intrahepatic cholangiocarcinoma; pCCA, Perihilar cholangiocarcinoma; dCCA, Extrahepatic cholangiocarcinoma; UICC 1–4, Union for International Cancer Control stages 1–4; pT, Histopathologic tumor stage evaluation; pN, Histopathologic lymph node evaluation; M, Distant metastases; G, Grade of differentiation; R, Resection margins; L/V, Invasion into lymphatic vessels or veins; Pn, Perineural invasion; HBV, Hepatitis B virus; HCV, Hepatitis C virus; PSC, Primary sclerosing cholangitis.
